# Synergistic Effects of Vermicompost and Biochar Amendments on Soil Fertility and Wheat Productivity in Bangladesh Floodplain Soils

**DOI:** 10.1155/sci5/6624984

**Published:** 2024-12-04

**Authors:** Ahmed Khairul Hasan, Shams Shaila Islam, Marina Jahan, Sinthia Afsana Kheya, Md. Romij Uddin, Md. Shafiqul Islam, Thanet Khomphet

**Affiliations:** ^1^Department of Agronomy, Bangladesh Agricultural University, Mymensingh 2202, Bangladesh; ^2^Department of Agronomy, Hajee Mohammad Danesh Science & Technology University, Dinajpur 5200, Bangladesh; ^3^Department of Agricultural Technology, School of Agricultural Technology and Food Industry, Walailak University, Nakhon Si Thammarat 80160, Thailand

**Keywords:** biochar, mineral fertilizer, organic amendments, soil fertility, vermicompost, wheat yield

## Abstract

Biochar is gaining importance due to its potential to enhance soil health, crop yield, and quality. It may also promote more sustainable farming methods. This study evaluated the combined effects of biochar, vermicompost, and inorganic fertilizers on soil characteristics, growth, and yield in wheat. Ten different treatments were applied to wheat (cultivar BARI Gom-33). The tallest plants, highest total dry weight, and largest leaf area index were observed in plots where chemical fertilizers, rice husk biochar, poultry manure, and vermicompost were applied together. At harvest, the treatment containing 1/4 recommended fertilizer dose (RFD) + 1/4 poultry manure biochar + 1/4 rice husk biochar + 1/4 vermicompost produced the best yield and yield-contributing factors. The combination of biochar, vermicompost, and inorganic fertilizers increased grain production by 43.23%–79.48% compared with the control. These treatments also improved soil health by increasing available phosphorus, organic matter, carbon-to-nitrogen ratio, and organic carbon. In conclusion, the combined application of 1/4 RFD, 1/4 poultry manure biochar, 1/4 rice husk biochar, and 1/4 vermicompost can replace the sole use of chemical fertilizers and serve as a key component for sustainable crop production.

## 1. Introduction

Wheat (*Triticum aestivum* L.) is recognized worldwide as a crucial and nutritionally valuable grain for feed, providing essential minerals, carbohydrates, and protein [1]. Soil fertility significantly influences wheat productivity, and the reliance on synthetic fertilizers combined with the lack of organic inputs is degrading agricultural soil fertility [2]. Nitrogen (N) fertilizers, in particular, reduce soil organic carbon (SOC), and improper fertilization practices further decrease soil fertility [3–5]. Consequently, wheat production in Bangladesh has been adversely affected by declining soil fertility. Approximately 50% of the net agricultural land in Bangladesh contains less than 1.5% organic materials [6]. This low level of soil organic matter raises significant concerns about the sustainability of both agricultural productivity and soil health.

The traditional agricultural practices of applying chemical fertilizers can have numerous negative effects, including a severe impact on soil pH due to the elemental composition of these fertilizers. To achieve high yields, it is essential to enhance soil health with both organic matter and fertilizers. While the use of mineral fertilizers is necessary, they must be supplemented or replaced with readily available organic resources due to rising costs and concerns regarding soil health, environmental sustainability, and human health. Organic amendments combined with chemical fertilizers can improve soil fertility, crop growth, yield components, and root health [7]. Utilizing organic amendments such as biochar in combination with vermicompost can significantly enhance soil fertility and sustainably increase crop yield.

Biochar, also known as charred biomass or black carbon, is an organic material produced by pyrolyzing organic biomass, including logs, agricultural residues, animal dung, poultry manure, and urban waste at a temperature between 300 and 1000°C under partially or completely anaerobic conditions [8, 9]. Biochar may be produced from a variety of sources. Because of their natural availability, agricultural and forest biomasses are most frequently employed for the manufacture of biochar [10]. For nearly 2 decades, biochar has gained attention in agriculture because of its ability to enhance soil health, sequester carbon, aid in biosorption, treat wastewater, and support green innovations. Incorporating biochar into the soil has demonstrated positive effects on enhancing soil structure, increasing nutrient retention, promoting microbial growth, and improving plant nutrient uptake, growth, and yield [11–13]. Biochar is frequently criticized for having poor nutrient and mineral release capacities, even though it has many potential uses. Therefore, biochar should be applied in combination with either organic or inorganic fertilizers, or both, to improve soil quality and promote plant development [14].

Vermicomposting is increasingly used to create organic fertilizers from waste, as it is a straightforward biotechnological composting technique that is both userfriendly and environmentally sustainable [15]. The microbial composting of organic wastes is aided by earthworms, resulting in the production of organic fertilizer that is richer in organic matter, organic carbon, total and accessible N, phosphorus (P), and potassium (K) and increased microbial and enzyme activity. Vermicomposting, according to Kaur [16], is an aerobic biological process that can be controlled to produce biodegradable humus from organic waste, which makes it an appropriate method for managing soil amendments. Vermicompost is rich in plant nutrients, notably N, P, K, and sulfur (S). When treated with vermicompost, soils exhibit lower pH levels and higher amounts of organic materials, essential nutrients, and dissolved salts [17]. Organic fertilizers are essential to sustainable agriculture because they preserve nutrient availability, enhance soil organic matter, and improve the physical and chemical characteristics of the soil, all of which lead to increased crop productivity. To increase crop production, soil fertility, plant nutrition, and overall agricultural sustainability, agricultural enterprises are using organic additions like compost and biochar [18, 19].

Consequently, it is imperative to put into action a thorough plan that incorporates the coordinated use of fertilizers. In this context, we present an experimental investigation to assess the effects of biochar and vermicompost on soil properties, wheat growth, and yield. In recent years, organic amendments such as biochar and vermicompost have gained significant attention for their role in enhancing soil fertility and improving crop productivity. Biochar, in particular, has been extensively studied for its ability to improve soil structure, increase water retention, and sequester carbon, thereby contributing to environmental sustainability. However, while it shows promise in improving soil physical properties, several studies have reported limitations in its ability to provide sufficient nutrients to crops without supplementary fertilization. To address this limitation, researchers have suggested combining biochar with organic or inorganic fertilizers to enhance nutrient availability and improve crop yield [14]. Research focusing on the combined use of biochar and vermicompost as a soil amendment strategy is still limited, particularly in the context of floodplain soils, which are common in Bangladesh and present unique challenges in terms of nutrient leaching and soil fertility maintenance.

While past studies have explored the individual effects of biochar and vermicompost on soil health and crop productivity, there is a paucity of research examining the synergistic effects of combining these two organic amendments. The majority of research focus on biochar's role in improving soil health [8, 20–23] or vermicompost's [24] ability to increase the nutrient content, but few have investigated [25–27] the cumulative effect of biochar and vermicompost on wheat growth and yield, particularly in the context of Bangladesh's floodplain soils. These soils are prone to nutrient loss and organic matter depletion due to flooding and intensive farming practices, making the optimization of soil amendment strategies crucial for sustainable wheat production. This study addresses the research gap by evaluating the combined effects of biochar and vermicompost on soil properties, wheat growth, and yield in a floodplain soils. By focusing on the synergistic impact of these two organic amendments in a challenging agricultural context, our research provides novel insights that could contribute to more sustainable agricultural practices in Bangladesh and other regions with similar soil conditions.

## 2. Materials and Methods

### 2.1. Location, Climate, and Soil

**Figure 1:**
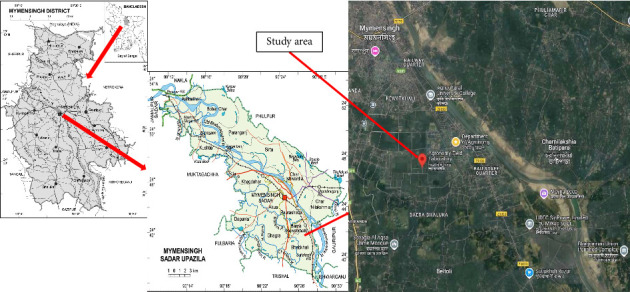
Map of Mymensingh Sadar Upazila road network showing study sites. *Source*: Google map.

From October 2019 to April 2020, research was conducted at the experimental field, Bangladesh Agricultural University, Mymensingh. The experimental area is in the Old Brahmaputra floodplain (AEZ-9) (Figure 1), characterized by a humid subtropical monsoon climate. The climatic conditions during the research period are detailed in Table 1. The soils in this region are predominantly silty loam, dark gray in color, low in organic matter, and exhibit low overall fertility. The soil chemical composition at the beginning stage from the field is shown in Table 2.

### 2.2. Experimental Setup

Along with the recommended fertilizer dose, three types of compost, for example, vermicompost, rice husk biochar, and poultry liter biochar were utilized in the experiment. BARI Gom-33 wheat variety was used as the test crop. BARI Gom-33 was chosen for this study because of its tolerance to Bangladesh's different climatic conditions, high production potential of 4.0–5.0 tons per hectare, and favorable reaction to organic fertilizers. Developed by the Bangladesh Agricultural Research Institute, this wheat type is well-suited for local agriculture, contributing considerably to food security and farmer livelihoods. BARI Gom-33 is also disease resistant, making it an excellent choice for increasing yield.

The research featured nine treatments, viz., T0 = no fertilizer, T1 = recommended fertilizer dose (RFD), T2 = 1/2 RFD + 1/2 poultry manure biochar, T3 = 1/2 RFD + 1/2 rice husk biochar, T4 = 1/2 RFD + 1/2 vermicompost, T5 = 1/3 RFD + 1/3 poultry manure biochar + 1/3 vermicompost, T6 = 1/3 RFD + 1/3 rice husk biochar + 1/3 vermicompost, T7 = 1/3 RFD + 1/3 poultry manure biochar + 1/3 rice husk biochar, T8 = 1/4 RFD + 1/4 poultry manure biochar + 1/4 rice husk biochar + 1/4 vermicompost, and T9 = 1/3 poultry manure biochar + 1/3 rice husk biochar + 1/3 vermicompost. The experiment was laid out in a randomized complete block design with three replications. The experimental field was divided into three blocks to represent the replication. Each block was then divided into 10-unit plots with raised bunds to accommodate 10 treatment combinations. Thus, the total number of plots was 30. The area of each plot was 3 m^2^ (2 m × 1.5 m). Treatments were randomly distributed in unit plots.

This experiment's nine treatment combinations are strategically planned to assess the effects of various organic amendments and fertilizer dosages on wheat development, addressing the research subject of optimizing nutrient management for increased crop output and soil health. The treatments vary from a control (no fertilizer) to a RFD, as well as various combinations of RFD and organic additions such as poultry manure biochar, rice husk biochar, and vermicompost. These combinations are designed to evaluate the advantages of incorporating organic materials, increasing nutrient availability, and improving soil structure while lowering dependency on chemical fertilizers. The randomized complete block design, which includes three replications and properly sized plots, reduces environmental variability and enables valid comparisons. This holistic method seeks to uncover effective nutrient management solutions that encourage sustainable agricultural practices and increase productivity in Bangladesh, while also giving useful insights into optimizing fertilizer use and improving soil health.

### 2.3. Selection of Organic Amendments

Three organic amendments—vermicompost, rice husk biochar, and poultry litter biochar—were carefully chosen for their individual nutritional profiles and complementing benefits to soil health and plant development. Vermicompost contains nutrients (10.8 g·kg^−1^ total N, 1020 mg·kg^−1^ accessible P) and beneficial microbes that promote nutrient cycling, improve soil structure, and enhance moisture retention due to its high organic carbon content (75.7 g·kg^−1^). Rice husk biochar contains 337.6 g·kg^−1^ of organic carbon and a balanced nutritional profile (18.1 g·kg^−1^ N, 1149 mg·kg^−1^ P). Its porous structure aids in nutrient retention and its neutral pH (7.5) supports good nutrient availability. Poultry litter biochar has the largest nutritional content (30.8 g·kg^−1^ N, 1437 mg·kg^−1^ P) and significant S (2094 mg·kg^−1^), which are critical for plant health. It also has a high cation exchange capacity (35.68 cmolc·kg^−1^) for efficient nutrient retention (Table 3). These supplements address acute nutritional deficits, increase microbial activity, and improve soil structure, making them necessary for sustainable agricultural methods.

### 2.4. Production and Characterization of Biochar

Biochar feedstocks include agricultural waste, wood-based residues, municipal waste, algae, livestock waste, wastewater, sewage sludge, and biosolids. In this experiment, rice husk and poultry litter were used as feedstocks to produce biochar. A cylindrical drum was employed to minimize pyrolysis heat loss. The kiln measured 2.5 m in length and 2 m in diameter, featured a chimney, and was coated with rough iron sheets. The drum had 20 aeration holes. The feedstock was placed into an iron drum for pyrolysis, which measured 100 cm in length and 60 cm in diameter and had a nearly airtight lid. A digital temperature recorder was inserted into the kiln through an aeration hole to record the pyrolysis temperature at 30-min intervals. The final land preparation involved spreading both types of biochars.

### 2.5. Crop Husbandry

The land preparation began with two ploughings using a power tiller, followed by additional ploughing, cross-ploughing, and leveling with a ladder. After leveling, the experimental plots were established according to the chosen treatments and design. The recommended fertilizer dosage was 220 kg urea, 180 kg triple superphosphate (TSP), 50 kg muriate of potash (MoP), and 100 kg gypsum per hectare [28]. The entire amounts of TSP, MoP, and gypsum were applied during final land preparation by mixing with the soil and spading as per the treatment specifications. Urea was top-dressed in two splits at 17 and 35 days after sowing (DAS), according to the treatment requirements. On November 20^th^, wheat seeds were sown in deep furrows created by hand raking, maintaining a spacing of 25 cm between rows, at 120 kg·ha^−1^, to ensure successful germination and facilitate intercultural operations. Three hand weeding were performed: one at 20 days, one at 40 days, and one at 60 days after seeding. Thinning and gap filling were carried out as needed. Irrigated water was provided at the crown root initiation (CRI) stage, 20 days after seeding. The crop was harvested on March 17, 2020, when 90% of the grains had turned golden yellow. The crop from each plot was collected and transported to the threshing floor in individual bundles with appropriate tags. The grain yields and straw weight were measured after proper sun-drying to a constant weight.

### 2.6. Soil Analysis

Soil samples were randomly collected from experimental field at the beginning and during the time of harvesting of wheat for chemical analysis. The samples were supplied to the Soil Resource Development Institute (SRDI) of Mymensingh, Bangladesh for analysis.

### 2.7. Data Record

Data on growth parameters (plant height, leaf area index, number of total tillers hill^−1^, and total dry matter), yield, and yield components were recorded from the sample plots. From randomly chosen sample plants from each plot, data of yield and yield-contributing traits were recorded.

### 2.8. Biological Yield

Biological yield refers to the total biomass produced by the wheat crop, including both grain and straw.

(1) $$\begin{array}{c} \text{Biological yield}=\text{grain yield}+\text{straw yield}. \end{array}$$

### 2.9. Harvest Index (HI)

HI is a measure of crop efficiency in allocating biomass to the grain and is calculated using the following formula:

(2) $$\begin{array}{c} \text{HI }\left(\%\right)=\left(\frac{g\text{rain }y\text{ield}}{b\text{iological }y\text{ield}}\right)\times 100. \end{array}$$

### 2.10. Carbon-to-Nitrogen (C:N) Ratio

The C:N ratio was estimated using the organic carbon and total nitrogen content. The C:N ratio was then estimated using the following formula:

(3) $$\begin{array}{c} C:N\text{ ratio}=\left(\frac{c\text{ardon content }\left(\%\right)}{n\text{itrogen content }\left(\%\right)}\right). \end{array}$$

### 2.11. Statistical Analysis

All the data (plant growth, yield, yield components, plant nutrient contents, and soil analysis after harvest) were analyzed using analysis of variance (ANOVA) using a computer package MSTAT-C program. The mean differences among the treatments were adjusted by Duncan's multiple range test (DMRT) [29].

## 3. Results

### 3.1. Climatic Data and Its Influence on Wheat Growth and Compost Treatments

During the research period, the maximum, lowest, and mean temperatures ranged from 12.85°C to 29.50°C, all of which are ideal for wheat development. Wheat, a cool-season crop, thrives at such mild temperatures, especially in the early vegetative development phases. The lowest mean temperature in January (19.40°C) certainly aided tillering, which requires lower temps. Temperatures rose somewhat in February and March (up to 23.30°C), perhaps promoting grain growth and filling. From November to February, rainfall was low, ranging between 0 and 1.00 mm, necessitating supplementary irrigation to avoid water stress during wheat's vital development phases. Rainfall in March (163.70 mm) may have aided grain filling (Table 1).

However, significant rainfall may have increased the danger of waterlogging, which might have an impact on root health and nutrient absorption, particularly in regions where organic amendments such as vermicompost and biochar were applied. The relative humidity fluctuated between 74.71% in February and 81.40% in December. Moderate humidity levels like this are typically beneficial for wheat development since they help to maintain appropriate moisture conditions for both the crop and the microbial activity required for compost decomposition. The moderate humidity most likely increased the efficiency of vermicompost in releasing nutrients into the soil while ensuring that the soil kept enough moisture for plant development. Sunshine hours decreased progressively throughout the months, from 204.80 in November to 151.60 in March (Table 1).

Wheat growth benefits from enough sunshine, especially during the grain-filling stage. The increased daylight hours during the early months of the crop cycle most likely aided photosynthesis and plant vigor. The decrease in sunshine in March may have had a little impact on the pace of grain filling, but the earlier stages supplied adequate light for appropriate crop establishment and growth. Irrigation was required between November and February because of the low rainfall and high sunlight hours, allowing the biochar and vermicompost additions to properly interact with the soil. Vermicompost, which enhances soil organic matter, may have benefited from the mild temperatures and humidity that encouraged microbial activity and nutrient availability in the early vegetative phases. Heavy rainfall in March might have caused nutrient leaching. Biochar's water retention qualities may have offset the detrimental impacts of waterlogging by improving root health and reducing nutrient loss, maintaining crop development during the vital grain-filling stage.

### 3.2. Initial Soil Characteristics

The sandy loam soil has low organic matter (5.3 g·kg^−1^), low N (0.6 g·kg^−1^), low P (12.39 mg·kg^−1^), low K (10.50 mg·kg^−1^), and extremely low S (0.125 mg·kg^−1^), indicating significant nutrient deficiencies hindering plant growth. The ideal pH is 6.61, however the low cation exchange capacity (10.52 cmolc·kg^−1^) and moderate moisture levels (17.54%) make nutrient retention and availability more challenging (Table 2). Utilizing organic amendments such as vermicompost and biochar is crucial in this situation. These supplements can boost organic matter content, nutrient availability, cation exchange capacity, beneficial microbial activity, and water retention, relieving early soil inadequacies while also supporting sustainable farming operations.

### 3.3. Growth Parameters

**Figure 2:**
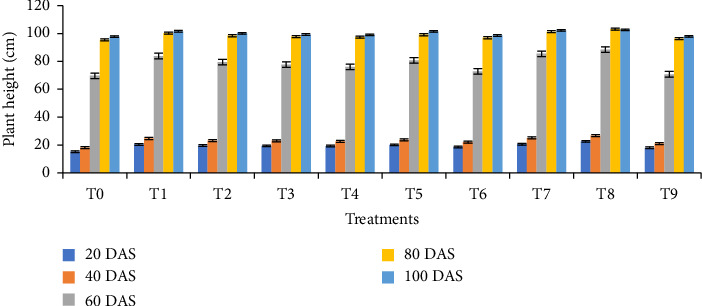
Effect of biochar and its combination with vermicompost on plant height of wheat at different days after sowing. T0 = no fertilizer; T1 = RFD; *T*2 = 1/2 RFD + 1/2 poultry manure biochar; T3 = 1/2 RFD + 1/2 rice husk biochar; T4 = 1/2 RFD + 1/2 vermicompost; T5 = 1/3 RFD + 1/3 poultry manure biochar + 1/3 vermicompost; T6 = 1/3 RFD + 1/3 rice husk biochar + 1/3 vermicompost; T7 = 1/3 RFD + 1/3 poultry manure biochar + 1/3 rice husk biochar; T8 = 1/4 RFD + 1/4 poultry manure biochar + 1/4 rice husk biochar + 1/4 vermicompost; and T9 = 1/3 poultry manure biochar + 1/3 rice husk biochar + 1/3 vermicompost. Error bars indicate standard error.

**Figure 3:**
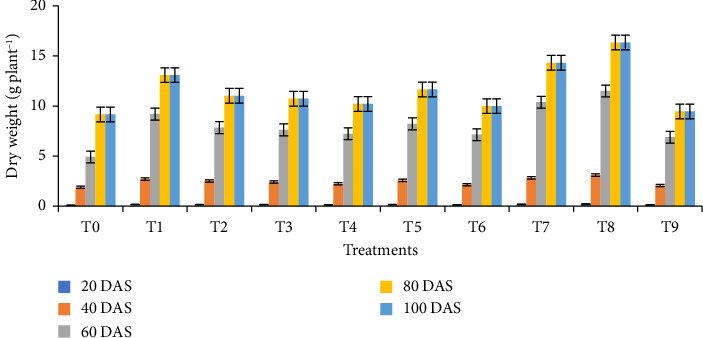
Effect of biochar and its combination with vermicompost on dry weight of wheat at different days after sowing. T0 = No fertilizer; T1 = RFD; T2 = 1/2 RFD + 1/2 poultry manure biochar; T3 = 1/2 RFD + 1/2 rice husk biochar; T4 = 1/2 RFD + 1/2 vermicompost; T5 = 1/3 RFD + 1/3 poultry manure biochar + 1/3 vermicompost; T6 = 1/3 RFD + 1/3 rice husk biochar + 1/3 vermicompost; T7 = 1/3 RFD + 1/3 poultry manure biochar + 1/3 rice husk biochar; T8 = 1/4 RFD + 1/4 poultry manure biochar + 1/4 rice husk biochar + 1/4 vermicompost; and T9 = 1/3 poultry manure biochar + 1/3 rice husk biochar + 1/3 vermicompost. Error bars indicate standard error.

**Figure 4:**
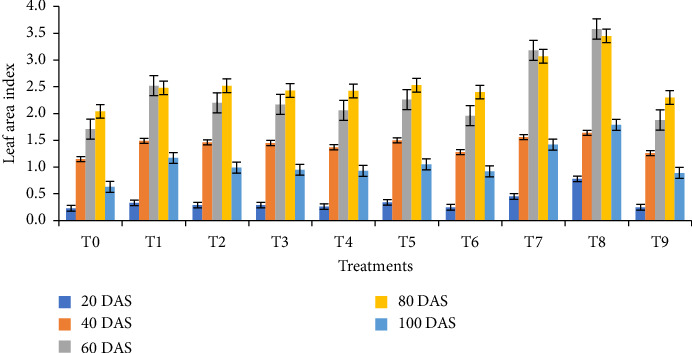
Effect of biochar and its combination with vermicompost on leaf area index of wheat at different days after sowing. T0 = No fertilizer; T1 = RFD; T2 = 1/2 RFD + 1/2 poultry manure biochar; T3 = 1/2 RFD + 1/2 rRice husk biochar; T4 = 1/2 RFD + 1/2 vermicompost; T5 = 1/3 RFD + 1/3 poultry manure biochar + 1/3 vermicompost; T6 = 1/3 RFD + 1/3 rice husk biochar + 1/3 vermicompost; T7 = 1/3 RFD + 1/3 poultry manure biochar + 1/3 rice husk biochar; T8 = 1/4 RFD + 1/4 poultry manure biochar + 1/4 rice husk biochar + 1/4 vermicompost; and T9 = 1/3 poultry manure biochar + 1/3 rice husk biochar + 1/3 vermicompost. Error bars indicate standard error.

**Figure 5:**
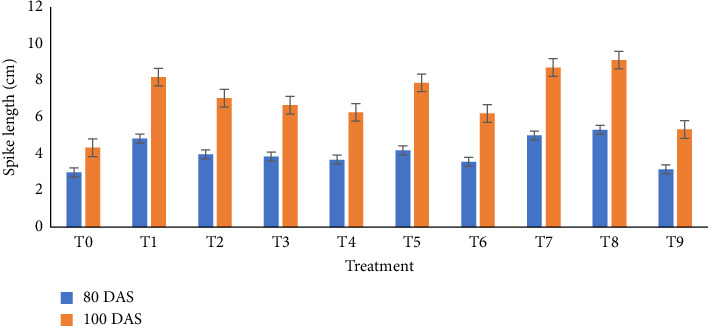
Effect of biochar and its combination with vermicompost on spike length o of wheat at different days after sowing. T0 = no fertilizer; T1 = RFD; T2 = 1/2 RFD + 1/2 poultry manure biochar; T3 = 1/2 RFD + 1/2 rice husk biochar; T4 = 1/2 RFD + 1/2 vermicompost; T5 = 1/3 RFD + 1/3 poultry manure biochar + 1/3 vermicompost; T6 = 1/3 RFD + 1/3 rice husk biochar + 1/3 vermicompost; T7 = 1/3 RFD + 1/3 poultry manure biochar + 1/3 rice husk biochar; T8 = 1/4 RFD + 1/4 poultry manure biochar + 1/4 rice husk biochar + 1/4 vermicompost; and T9 = 1/3 poultry manure biochar + 1/3 rice husk biochar + 1/3 vermicompost. Error bars indicate standard error.

The impact of biochar and its combination with vermicompost significantly affected plant height, leaf area index, and dry weight per plant at 20, 40, 60, 80, and 100 DAS. At all sampling dates, plants cultivated under the T8 treatment (1/4 RFD + 1/4 poultry manure biochar + 1/4 rice husk biochar + 1/4 vermicompost) exhibited the maximum heights of 22.59 cm, 26.72 cm, 88.44 cm, 103.11 cm, and 102.67 cm, respectively (Figure 2). In addition, this treatment demonstrated the highest dry weight per plant at 20 DAS (0.21 g), 40 DAS (3.10 g), 60 DAS (11.50 g), 80 DAS (16.35 g), and 100 DAS (13.95 g) (Figure 3). Similarly, the highest leaf area index readings for the T8 treatment were observed at 20, 40, 60, 80, and 100 DAS, being 0.78, 1.64, 3.58, 3.45, and 1.79, respectively (Figure 4). Furthermore, the combination of biochar and vermicompost significantly influenced spike length at 80 and 100 DAS. The T8 treatment resulted in the longest spikes, measuring 5.31 cm at 80 DAS and 9.11 cm at 100 DAS (Figure 5). In contrast, plants grown under control conditions exhibited the minimum plant height, leaf area index, dry weight per plant, and spike length at all sampling dates.

### 3.4. Yield and Yield-Contributing Traits

With the exception of noneffective tillers per hill and sterile spikelets per spike, biochar and its combination with vermicompost had a substantial impact on all yield-contributing traits and the yield of wheat. At the harvesting stage, plant height ranged from 98.80 cm to 105.70 cm, with the combination of 1/4 RFD + 1/4 poultry manure biochar + 1/4 rice husk biochar + 1/4 vermicompost treatment exhibiting the greatest plant height, while the control treatment showed the minimum plant height. The trial results indicated that plants grown under the T8 treatment showed the maximum number of total tillers per hill (3.73), which is 12% higher than both the control (3.33) and the combination of ⅓ poultry manure biochar + 1/3 rice husk biochar + 1/3 vermicompost (3.33) treatments. The number of effective tillers per hill ranged from 2.40 to 3.40, with a 41.67% increase in effective tillers per hill documented in the T8 treatment compared with the control.

The longest spike (13.65 cm) was observed in the T8 treatment while the shortest (12.62 cm) was in the control treatment. The number of spikelets per spike varied from 15.68 to 16.81, with the highest number recorded in the T8 treatment and the lowest number in the control (T0) treatment. Grains per spike increased from 41.14 in the control treatment to 47.53 with the application of 1/4 RFD + 1/4 poultry manure biochar + 1/4 rice husk biochar + 1/4 vermicompost. The 1000-grain weight ranged from 47.20 g to 47.53 g, with the T8 treatment yielding the maximum weight, while the control (T0) and T9 treatments produced the lowest weights.

**Figure 6:**
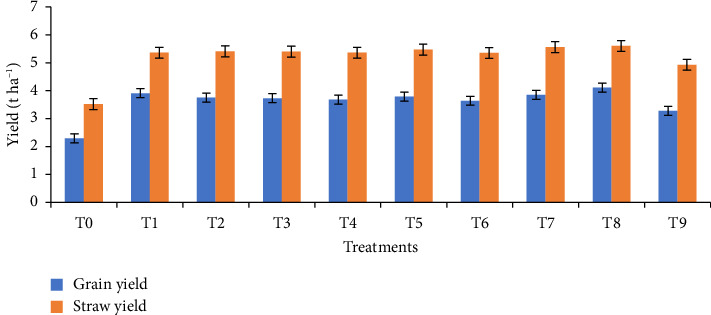
Yield of wheat as affected by different biochar and vermicompost combinations. T0 = no fertilizer; T1 = RFD; T2 = 1/2 RFD + 1/2 poultry manure biochar; T3 = 1/2 RFD + 1/2 rice husk biochar; T4 = 1/2 RFD + 1/2 vermicompost; T5 = 1/3 RFD + 1/3 poultry manure biochar + 1/3 vermicompost; T6 = 1/3 RFD + 1/3 rice husk biochar + 1/3 vermicompost; T7 = 1/3 RFD + 1/3 poultry manure biochar + 1/3 rice husk biochar; T8 = 1/4 RFD + 1/4 poultry manure biochar + 1/4 rice husk biochar + 1/4 vermicompost; and T9 = 1/3 poultry manure biochar + 1/3 rice husk biochar + 1/3 vermicompost. Error bars indicate standard error.

The highest grain yield (4.11 t·ha−1) was achieved with the 1/4 RFD + 1/4 poultry manure biochar + 1/4 rice husk biochar + 1/4 vermicompost treatment, whereas the control treatment generated the lowest grain yield (2.29 t·ha−1). Grain yield increased by 43.23%–79.48% due to the application of biochar and its combination with vermicompost. Similarly, the T8 treatment resulted in the highest straw yield (5.60 t·ha^−1^), which is 59.09% higher than the control (3.52 t·ha^−1^) (Figure 6). Biological yield increased by 41.31%–67.13% compared with the control, with the highest biological yield (9.71 t·ha^−1^) recorded in the T8 treatment and the lowest (5.81 t·ha−1) in the control (T0) treatment. The highest HI (42.33%) was also registered with the T8 treatment, while the lowest (39.45%) was obtained from the control treatment **(**Table 4).

### 3.5. Postharvest Soil Properties

The soil available P content ranged between 14.52 ppm and 17.06 ppm across the treatments. The treatment consisting of 1/4 RFD + 1/4 poultry manure biochar + 1/4 rice husk biochar + 1/4 vermicompost exhibited the highest P content at 16.42 ppm, which was statistically similar to the P levels in treatments T1 (16.31 ppm), T5 (16.16 ppm), and T6. The lowest P availability was observed in the control treatment. Significant effects of the treatments were also noted for K and S availability. The T1 treatment (recommended fertilizer dose) demonstrated the highest K availability (0.12 meq/100 g) and S availability (16.57 ppm). Conversely, the lowest K and S availability (0.09 meq/100 g and 8.14 ppm, respectively) were recorded in the control treatment. The total N content of the soil varied significantly among treatments, ranging from 0.08% to 0.11%. The highest N (0.11%) was observed in treatments T1 (recommended fertilizer dose) and T5 (1/3 RFD + 1/3 poultry manure biochar + 1/3 vermicompost), while the lowest nitrogen (0.08%) was found in T0. The soil organic matter ranged from 1.51% to 2.50%. Treatment T8 (1/4 RFD + 1/4 poultry manure biochar + 1/4 rice husk biochar + 1/4 vermicompost) showed the highest level of organic matter, whereas the lowest organic matter content was recorded in the control treatment. The highest percentage of organic carbon (1.44%) was observed in treatment T8. The C:N ratio of the soil varied from 10.04 to 14.44, with the highest ratio found in the treatment comprising 1/4 RFD + 1/4 poultry manure biochar + 1/4 rice husk biochar + 1/4 vermicompost and the C:N lowest ratio in the control treatment (Table 5).

### 3.6. Principal Component Analysis (PCA)

**Figure 7:**
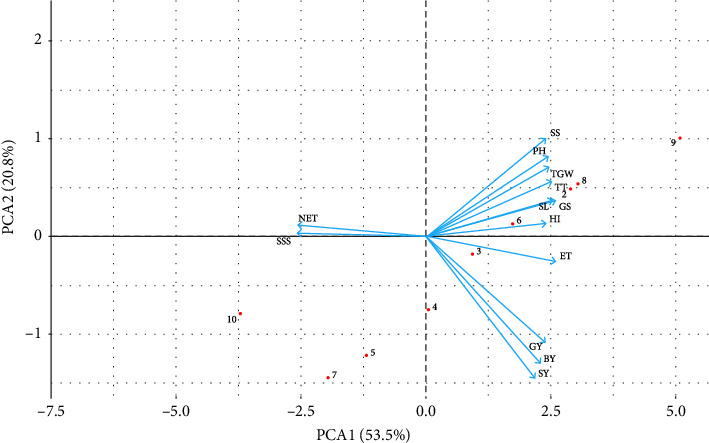
Biplot of principal component analysis. For PCA, data on weed growth and yield and yield contributing attributes under residues treatment. The red dots are indicating treatments like T0 = no fertilizer; T1 = RFD; T2 = 1/2 RFD + 1/2 poultry manure biochar; T3 = 1/2 RFD + 1/2 rice husk biochar; T4 = 1/2 RFD + 1/2 vermicompost; T5 = 1/3 RFD + 1/3 poultry manure biochar + 1/3 vermicompost; T6 = 1/3 RFD + 1/3 rice husk biochar + 1/3 vermicompost; T7 = 1/3 RFD + 1/3 poultry manure biochar + 1/3 rice husk biochar; T8 = 1/4 RFD + 1/4 poultry manure biochar + 1/4 rice husk biochar + 1/4 vermicompost; and T9 = 1/3 poultry manure biochar + 1/3 rice husk biochar + 1/3 vermicompost. BY = biological yield (t ha^−1^), ET = effective tillers hill^−1^, GS = grains spike^−1^ (no.), GY = grain yield (t ha^−1^), HI = harvest index (%), NET = Non-effective tillers hill^−1^, PH = plant height (cm), SL = spike length (cm), SS = spikelets spike^−1^ (no.), SSS = sterile spikelets spike^−1^ (no.), SY = straw yield (t ha^−1^), TGW = 1000-grain weight (g), TT = total tillers hill^−1^.

PCA was used to determine the traits that best describe the combined effects of biochar, vermicompost, and inorganic fertilizers on soil characteristics, growth, and yield in wheat. The biplot of the first two principal components and the loadings of variables are presented in Figure 7. The *x*-axis represents the first principal component (PC1), which accounts for 53.5% of the total variance and the *y*-axis represents the second principal component (PC2), which accounts for 20.8% of the total variance. The ET, HI, GS, SL, TT, TWS, and PH variables have a strong positive correlation with PC1. The NET and SSS variables have a strong positive correlation with PC2. The GY, BY, SY, and SS variables have a lower contribution to both PC1 and PC2 as they are near to the origin. The ET, HI, GS, SL, TT, TWS, and PH variables are closely aligned, indicating a strong positive correlation with each other. Treatments T3 and T4 are strongly influenced by PC2 and are distinct from other observations, and treatments T1, T2, T5, T7, and T8 are strongly influenced by PC1 and are distinct from other observations.

### 3.7. Correlation Analysis

**Figure 8:**
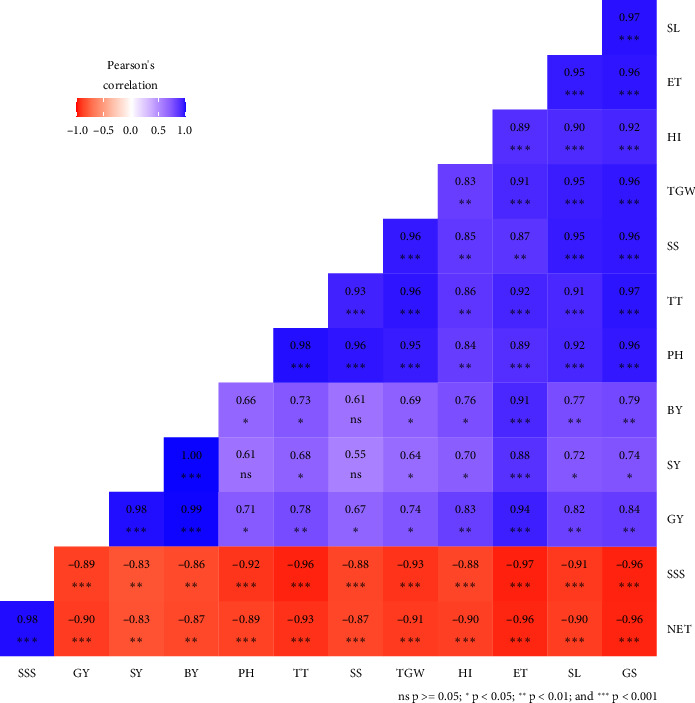
Pearson correlation analysis of yield-contributing traits of wheat under the combined application of biochar, vermicompost, and inorganic fertilizers. BY = biological yield (t ha^−1^), ET = effective tillers hill^−1^, GS = grains spike^−1^ (no.), GY = grain yield (t ha^−1^), HI = harvest index (%), NET = Non-effective tillers hill^−1^, PH = plant height (cm), SL = spike length (cm), SS = spikelets spike^−1^ (no.), SSS = sterile spikelets spike^−1^ (no.), SY = straw yield (t ha^−1^), TGW = 1000-grain weight (g), TT = total tillers hill^−1^. ⁣^∗∗∗^, ⁣^∗∗^, ⁣^∗^, and ns represent probability of *p* < 0.001, < 0.01, < 0.05 and ≥ 0.05, respectively.

A correlation was performed to identify the interrelationship among different yield traits and yield of wheat influenced by combined effects of biochar, vermicompost, and inorganic fertilizers (Figure 8). The analysis revealed that the SL had a strong positive correlation (*p* < 0.001) with the GS. ET had a strong positive correlation with the GS and SL. HI also had a strong positive correlation with the GS, SL, and ET. TGW had a strong positive correlation with the GS, SL, and ET and a moderate positive correlation with the HI. SS had a strong positive correlation with the GS, SL, and TGW and a moderate positive correlation with the HI and ET. TT had a strong positive correlation with the GS, SL, ET, TGW, and SS and a moderate positive correlation with the HI. PH had a strong positive correlation with the GS, SL, ET, TGW, SS, and TT and a moderate positive correlation with the HI. BY had a strong positive correlation with the ET a moderate positive correlation with the SL and GS and a positive correlation with HI, TGW, TT, and PH. SY had a strong positive correlation with the ET and BY and a positive correlation with SL, GS, HI, TGW, and TT. GY had a strong positive correlation with the ET, SY, and BY and a moderate positive correlation with the GS, SL, and TT and a positive correlation with TGW, SS, and PH. NET had a strong positive correlation with the SSS. So, this study revealed that the combined application of biochar, vermicompost, and inorganic fertilizers had a positive impact on wheat yield.

## 4. Discussion

The combination of biochar and compost offers several advantages over using either one alone when mixed with soil. These benefits include enhanced nutrient utilization, biological stimulation from biochar, increased nutrient availability due to biological nitrogen fixation, and reduced nutrient leaching [30]. The research demonstrated that incorporating inorganic fertilizers, vermicompost, and biochar into nutrient-deficient soil positively impacted wheat growth parameters. The combination of 1/4 RFD + 1/4 poultry manure biochar + 1/4 rice husk biochar + 1/4 vermicompost resulted in the highest plant height, leaf area index, and dry weight per plant. Plant height serves as an indicator of biomass production and growth rate. Alvarez et al. [31] found that biochar and vermicompost enhance nutrient availability, particularly P, which promotes root development and nutrient absorption, thereby increasing plant height. The bioavailability of minerals, in conjunction with plant hormones such as gibberellins and auxins, can influence plant height [32]. Similarly, previous investigations have reported that the use of biochar and vermicompost improves biomass production [33]. In addition, the application of biochar and vermicompost has been frequently associated with increases in leaf area and leaf number [31].

Both types of biochar, especially when combined with vermicompost, contain significant levels of N, P, calcium, and magnesium, which contribute to enhanced plant growth characteristics. Adding biochar to soil has been shown to enhance soil physical properties, such as decreasing bulk density, and increasing nutrient availability, thus promoting plant growth [34]. According to Cogger et al. [35], plant roots can more readily access nutrients in the rhizosphere, which supports plant growth. Active functional groups in biochar increase the cation exchange capacity of the soils and nutrient absorption ability of growing plants, leading to improved plant development.

The combination of inorganic and organic nutrient sources, known as integrated plant nutrient systems (IPNSs), significantly enhanced wheat production and yield-contributing characteristics compared with the use of fertilizers alone. Among the 10 treatments evaluated, the combination of 1/4 RFD + 1/4 poultry manure biochar + 1/4 rice husk biochar + 1/4 vermicompost yielded the highest grain output, surpassing all other organic and inorganic fertilizer combinations. However, biochar-fertilizer mixtures outperform pure fertilizers in terms of yield and plant nutrition [36]. Karer et al. [37] also found that using biochar with normal N fertilizer increased grain production by 10% compared with not using N. But the second-highest grain production was achieved with the recommended fertilizer dose alone. This indicates that organic amendments alone are insufficient for optimal yield; inorganic fertilizers are necessary for achieving satisfactory results.

The enhancement in grain yield can be responsible to improved yield parameters such as effective tillers per hill, number of spikelets per spike, spike length, number of grains per spike, and 1000-grain weight, which were superior in the combined application of biochar amendments, vermicompost, and inorganic fertilizers. The study revealed that the simultaneous application of fertilizers, biochar, and vermicompost positively impacted vegetative growth, thereby enhancing grain output. This improvement is due to the enhanced soil nutrient availability provided by the combination of biochar and compost. Biochar and vermicompost boost crop yields and soil health by increasing nutrient availability, water retention, and microbial activity. Biochar improves soil nutrient retention and lowers nitrogen leaching [21], whereas vermicompost offers easily accessible nutrients and encourages root growth. Both additives promote water retention: biochar through its porous structure, and vermicompost through increased soil aggregation. They also promote healthy soil bacteria, which help with nutrient cycling and root health. Together, these systems generate a resilient soil environment that promotes better yields and long-term soil fertility [38]. Increased tiller numbers, facilitated by adequate nutrition, are likely due to improved root growth and the enhanced translocation of carbohydrates from source to developing points [39].

These findings align with research by Manolikaki and Diamadopoulos [40], who observed improved plant development with the amendment of rice husk biochar and manures. Previous studies [41] have also shown that biochar application to coarse textured and degraded soils can boost crop output. Di et al. [42] found that the amendment of vermicompost to biochar amendments resulted in a significant increase in rice yield, ranging from 26.5% to 35.3%, compared with biochar alone. Similarly, Doan et al. [27] reported substantial improvements in maize performance with the combination of both biochar and vermicompost compared with control treatments.

Biochar accelerates nutrient cycling in the soil and enhances nutrient and water uptake due to its distribution, size, structure, and microspores. Chan et al. [43] highlighted that the large surface area of activated carbon increases cation exchange sites in soil, improving nutrient availability for crops. Vermicompost contributes significantly to soil fertility through its substantial content of N, P, and K. These nutrients become more available to plants as they are exchanged at soil exchange sites [44]. In addition, vermicompost promotes the growth of beneficial soil microbes, aids in mineralization, and enhances nutrient cycling. It improves soil aeration, aggregation, and water storage capacity, which are critical for the growth of soil aerobic microorganisms. Increased water availability due to improved aggregation also positively impacts agricultural output [45, 46]. Furthermore, chemical fertilizers contribute to plant growth by promoting dry matter accumulation, larger leaf size, and reducing direct evaporation from the soil surface [47]. Therefore, combining biochar, vermicompost, and chemical fertilizers may be a more effective strategy for improving wheat growth and yield. This study demonstrated that the co-application of both organic amendments and inorganic fertilizers positively impacted soil properties. Results indicated that the combination of 1/4 RFD + 1/4 poultry manure biochar + 1/4 rice husk biochar + 1/4 vermicompost had the most beneficial effect on P, organic matter, C:N ratio, and organic carbon contents. Conversely, for N, K, and S, the recommended fertilizer dose alone exhibited the most positive effect.

It was observed that integrated applications of various compost types resulted in a higher C:N ratio compared with single applications of chemical fertilizers. The addition of biochar and vermicompost enhanced soil organic matter and soil organic carbon levels, leading to improved soil structure by increasing porosity, moisture content, and infiltration rates, while reducing dispersion ratio and bulk density. This is consistent with findings from Zhang et al. [48], who reported that coapplication of biochar and vermicompost improved SOM and SOC, thereby reducing soil erosion by increasing and stabilizing soil aggregate size. The high carbon content of biochar and vermicompost likely contributes to the stability of soil aggregates. The observed increase in soil C:N ratio with the application of biochar and vermicompost aligns with the previous research by Liu et al. [49]. The C:N ratio is critical for determining the balance between N immobilization and mineralization in the soil. The enhanced capacity of vermicompost and biochar to retain nutrients, water, and organic matter explains their effectiveness. Vermicompost helps mitigate nutrient and nitrate loss through leaching, while biochar enhances soil water and nitrogen retention in the root rhizosphere.

Chemical fertilizers produced more easily accessible N, K, and S than organic supplements. Although inorganic fertilizers improve early N mineralization, they have been shown to reduce soil quality by inducing acidity and compaction [50, 51]. Fageria and Baligar [52] have described their combined application with organic materials for overcoming such difficulties. The combined application of organic manures and synthetic fertilizer can assist to maintain soil fertility and production [53–55]. Both biochar and vermicompost were shown to increase soil aggregate dispersion, water retention capacity, and soil compaction. While biochar and vermicompost may generate some greenhouse gases (GHGs), they typically create lower emissions than traditional fertilizers. Biochar can trap carbon in soil, lowering total GHG emissions by stabilizing carbon and minimizing nitrogen loss. Vermicompost also reduces GHG emissions by slowing nutrient release and promoting microbial populations that contribute in the nitrogen cycle [56, 57]. Conventional fertilizers can cause large emissions, especially N_2_O, as they release nutrients faster than plants require. Overall, biochar and vermicompost provide a more sustainable solution, improving carbon storage and soil health while reducing environmental impact. The use of biochar and vermicompost in agriculture has significant environmental and agronomic benefits, encouraging more sustainable practices.

These organic additions increase nutrient availability, allowing for less reliance on chemical fertilizers and reducing nutrient runoff and water pollution. Biochar increases soil structure, nutrient retention, and carbon sequestration, which contribute to climate change mitigation by storing carbon in the soil. Vermicompost promotes microbial activity and nutrient cycling, which improves soil vitality. Both biochar and vermicompost increase water-holding capacity, which benefits drought-prone areas and stabilizes soil structure to avoid erosion. By minimizing the requirement for synthetic inputs, these amendments promote biodiversity and contribute to sustainability by lowering greenhouse gas emissions from soil [38].

Integrating biochar and vermicompost into Bangladeshi floodplain agriculture might considerably help smallholder farmers by increasing sustainability and resilience. Subsidies and incentives would make these additions more inexpensive, lowering reliance on chemical fertilizers and improving soil quality. Training programs in local biochar and vermicompost production might help farmers make better use of available resources such as rice husks and organic waste. Community-based production units would ensure a consistent supply and lower costs, whereas national climate policies that promote these practices could increase biodiversity, reduce greenhouse gas emissions, and improve soil resilience to erosion and flooding, ultimately improving food security and livelihoods.

## 5. Conclusion

Different combinations of biochar and vermicompost had varying effects on growth and yield of wheat. Among the combinations tested, the integration of poultry litter biochar, rice husk biochar, and vermicompost with fertilizers demonstrated superior performance. Specifically, the treatment comprising 1/4 RFD **+** 1/4 poultry manure biochar + 1/4 rice husk biochar + 1/4 vermicompost emerged as the most effective practice for wheat cultivation. The observed improvements in soil parameters suggest that substituting chemical fertilizers with vermicompost and biochar can enhance soil health. Thus, it can be concluded that the combined application of biochar and vermicompost, alongside the recommended N, P, and K, represents a more effective strategy for improving wheat growth, yield, and soil properties. This study revealed that mixing biochar and vermicompost with fertilizers improves wheat growth and production, particularly in floodplain soils. However, many limits must be recognized. The geographic focus on floodplain soils may limit the application of our findings to other soil types and agroecological zones. Furthermore, the short length of the research limits our understanding of these amendments' long-term effects on soil health and crop output. To increase the reliability of our findings and build on our research, more research in numerous crucial areas is advised.

## Figures and Tables

**Table 1:** Monthly temperature, relative humidity, rainfall, and sunshine from November 2019 to March 2020 at the research area.

Times	Temperature (°C)			Rainfall (mm)	Relative humidity (%)	Sunshine (hrs)
	Max	Min	Mean			
November 2019	29.50	18.10	23.40	1.00	81.00	204.80
December 2019	27.50	14.60	21.10	0.00	81.40	180.30
January 2020	25.08	12.85	19.40	0.00	76.74	168.90
February 2020	28.30	15.81	22.08	0.20	74.71	157.20
March 2020	27.30	18.00	23.30	163.70	78.52	151.60

*Note:* Source: Weather Yard, Department of Irrigation and Water Management, Bangladesh Agricultural University, Mymensingh.

**Table 2:** Chemical characteristics of soil of the experimental field.

Characteristics	Status
Texture	Sandy loam
Soil organic carbon (g kg^−1^)	5.3
Total N (g kg^−1^)	0.6
Available P (mg kg^−1^)	12.39
Available K (mg kg^−1^)	10.50
Available S (mg kg^−1^)	0.125
pH	6.61
Cation exchange capacity (cmolc kg^−1^)	10.52
Moisture (%)	17.54
Bulk density (g cm^−3^)	1.33

*Note:* Source: results obtained from chemical analysis of the initial soil sample done in the Department of Soil Science, Bangladesh Agricultural University, Mymensingh.

**Table 3:** Chemical properties of organic amendments used in the experiment.

Organic amendments	Vermicompost	Poultry manure biochar	Rice husk biochar
Organic carbon (g kg^−1^)	75.7	337.6	175.2
Total nitrogen (g kg^−1^)	10.8	30.8	18.1
Available P (mg kg^−1^)	1020	1437	1149
Available K (mg kg^−1^)	4.99	22.61	15.99
Available S (mg kg^−1^)	377	2094	415
pH	7.7	8.5	7.5
CEC (cmolc kg^−1^)	11.83	35.68	19.54

**Table 4:** Yield and yield-contributing traits of wheat as influenced by different biochar and vermicompost combinations.

Biochar and vermicompost	Plant height (cm)	Total tillers hill^−1^ (no.)	Effective tillers hill^−1^ (no.)	Noneffective tillers hill^−1^ (no.)	Spike length (cm)	Spikelets spike^−1^ (no.)	Sterile spikelets spike^−1^ (no.)	Grains spike^−1^ (no.)	1000- grain weight (g)	Biological yield (t ha^−1^)	Harvest index (%)
T0	98.80^d^	3.33^b^	2.40^b^	0.67	12.62^b^	15.68^b^	2.09	41.14^b^	47.20^b^	5.81^f^	39.45^f^
T1	104.40^a-c^	3.67^ab^	3.13^ab^	0.40	13.25^ab^	16.45^ab^	1.63	45.86^ab^	47.40^ab^	9.27^bc^	42.16^a^
T2	103.00^a-d^	3.60^ab^	3.07^ab^	0.47	13.13^ab^	16.13^ab^	1.73	44.44^ab^	47.37^ab^	9.16^cd^	40.92^b^
T3	100.90^b-d^	3.53^ab^	3.00^ab^	0.47	13.02^ab^	15.93^ab^	1.75	43.85^ab^	47.37^ab^	9.13^cd^	40.85^bc^
T4	99.90^b-d^	3.47^ab^	2.93^ab^	0.53	12.98^ab^	15.78^ab^	1.82	43.37^ab^	47.27^ab^	9.04^d^	40.71^c^
T5	104.40^a-c^	3.67^ab^	3.13^ab^	0.47	13.15^ab^	16.22^ab^	1.67	45.00^ab^	47.40^ab^	9.26^bc^	40.91^b^
T6	99.70^cd^	3.40^ab^	2.87^ab^	0.53	12.87^ab^	15.76^ab^	1.90	43.12^ab^	47.23^ab^	8.99^d^	40.48^d^
T7	104.60^ab^	3.67^ab^	3.20^a^	0.40	13.32^ab^	16.56^an^	1.62	46.01^ab^	47.47^ab^	9.41^b^	40.94^b^
T8	105.70^a^	3.73^a^	3.40^a^	0.40	13.65^a^	16.81^a^	1.61	47.17^a^	47.53^a^	9.71^a^	42.33^a^
T9	99.10^d^	3.33^b^	2.73^ab^	0.60	12.86^ab^	15.68^b^	1.95	41.94^b^	47.20^b^	8.21^e^	39.98^e^
$S_{\overset{¯}{x}}$	0.83	0.05	0.09	0.03	0.09	0.13	0.05	0.60	0.04	0.35	0.27
*F*-test	⁣^∗^	⁣^∗^	⁣^∗^	ns	⁣^∗^	⁣^∗^	ns	⁣^∗^	⁣^∗^	⁣^∗∗^	⁣^∗^
CV (%)	2.45	10.03	14.34	12.45	5.54	5.13	7.40	5.65	5.28	1.22	0.26

*Note:* Means with the same letters within the same column do not differ significantly. ns, not significant difference. T0 = no fertilizer; T1 = RFD; T2 = 1/2 RFD + 1/2 poultry manure biochar; T3 = 1/2 RFD + 1/2 rice husk biochar; T4 = 1/2 RFD + 1/2 vermicompost; T5 = 1/3 RFD + 1/3 poultry manure biochar + 1/3 vermicompost; T6 = 1/3 RFD + 1/3 rice husk biochar + 1/3 vermicompost; T7 = 1/3 RFD + 1/3 poultry manure biochar + 1/3 rice husk biochar; T8 = 1/4 RFD + 1/4 poultry manure biochar + 1/4 rice husk biochar + 1/4 vermicompost; and T9 = 1/3 poultry manure biochar + 1/3 rice husk biochar + 1/3 vermicompost.

⁣^∗^ and ⁣^∗∗^Significant at *p* < 0.05 and 0.01, respectively.

**Table 5:** Effect of different biochar and vermicompost combinations on soil properties.

Biochar and vermicompost	Available P (ppm)	Available K (meq 100 g^−1^)	Available S (ppm)	Total N (%)	Organic matter (%)	Organic carbon (%)	C:N ratio
T0	14.52^d^	0.09^c^	8.14^d^	0.08^e^	1.51^e^	0.88^f^	10.81^e^
T1	16.42^ab^	0.12^a^	16.57^a^	0.11^a^	1.96^d^	1.13^e^	10.04^f^
T2	15.06^cd^	0.11^b^	8.81^d^	0.10^bc^	2.15^c^	1.24^d^	12.74^cd^
T3	14.88^cd^	0.11^b^	8.77^d^	0.09^d^	2.14^c^	1.24^d^	13.31^bc^
T4	15.75^bc^	0.11^b^	10.89^c^	0.10^bc^	2.36^b^	1.37^b^	13.26^bc^
T5	16.31^ab^	0.11^b^	12.13^b^	0.11^a^	2.32^b^	1.34^b^	12.21^d^
T6	16.16^ab^	0.11^b^	10.08^c^	0.10^bc^	2.34^b^	1.36^b^	13.16^bc^
T7	15.60^b-d^	0.11^b^	10.38^c^	0.10^bc^	2.30^b^	1.33^bc^	13.31^bc^
T8	17.06^a^	0.11^b^	15.84^a^	0.10^bc^	2.50^a^	1.44^a^	14.44^a^
T9	14.61^d^	0.11^b^	8.34^d^	0.09^d^	2.19^c^	1.27^cd^	13.62^b^
$S_{\overset{¯}{x}}$	0.27	0.01	0.95	0.01	0.09	0.05	0.42
*F*-test	⁣^∗∗^	⁣^∗∗^	⁣^∗∗^	⁣^∗∗^	⁣^∗∗^	⁣^∗∗^	⁣^∗∗^
CV (%)	3.78	3.92	5.38	3.32	2.65	3.20	4.66

*Note:* Means with the same letters within the same column do not differ significantly. T0 = no fertilizer; T1 = RFD; T2 = 1/2 RFD + 1/2 poultry manure biochar; T3 = 1/2 RFD + 1/2 rice husk biochar; T4 = 1/2 RFD + 1/2 vermicompost; T5 = 1/3 RFD + 1/3 poultry manure biochar + 1/3 vermicompost; T6 = 1/3 RFD + 1/3 rice husk biochar + 1/3 vermicompost; T7 = 1/3 RFD + 1/3 poultry manure biochar +1/3 rice husk biochar; T8 = 1/4 RFD + 1/4 poultry manure biochar + 1/4 rice husk biochar + 1/4 vermicompost; and T9 = 1/3 poultry manure biochar + 1/3 rice husk biochar + 1/3 vermicompost.

⁣^∗∗^Significant at *p* < 0.01.

## Data Availability

The data that support the findings of this study are available from the corresponding author upon reasonable request.
